# Malaria exposure drives both cognate and bystander human B cells to adopt an atypical phenotype

**DOI:** 10.1002/eji.201948473

**Published:** 2020-05-04

**Authors:** Racheal Aye, Henry J. Sutton, Eunice W. Nduati, Oscar Kai, Jedida Mwacharo, Jennifer Musyoki, Edward Otieno, Juliana Wambua, Philip Bejon, Ian A. Cockburn, Francis M. Ndungu

**Affiliations:** 1 Department of Biosciences, Centre for Geographical Medicine Research (Coast), Kenya Medical Research Institute, Nairobi, Kenya; 2 Department of Immunology and Infectious Disease, John Curtin School of Medical Research, The Australian National University, Canberra, Australia; 3 Nuffield Department of Medicine, Centre for Tropical Medicine and Global Health, University of Oxford, Oxford, United Kingdom

**Keywords:** B-cell memory, immunological memory, malaria, *Plasmodium*, tetanus toxoid

## Abstract

Atypical memory B cells (aMBCs) are found in elevated numbers in individuals exposed to malaria. A key question is whether malaria induces aMBCs as a result of exposure to Ag, or non-Ag-specific mechanisms. We identified Plasmodium and bystander tetanus toxoid (TT) specific B cells in individuals from areas of previous and persistent exposure to malaria using tetramers. Malaria-specific B cells were more likely to be aMBCs than TT-specific B cells. However, TT-specific B cells from individuals with continuous exposure to malaria were more likely to be aMBCs than TT-specific B cells in individuals from areas where transmission has ceased. Finally, sequences of BCRs specific for a blood stage malaria-Ag were more highly mutated than sequences from TT-specific BCRs and under strong negative selection, indicative of ongoing antigenic pressure. Our data suggest both persistent Ag exposure and the inflammatory environment shape the B-cell response to malaria and bystander Ags.

## Introduction

Malaria, caused by parasites of the genus *Plasmodium,* was responsible for the deaths of 405,000 people in 2018 [1]. A characteristic feature of the epidemiology of malaria is the fact that individuals who recover from infection do not develop sterilising immunity [2]. However, children continually exposed to malaria transmission develop anti-disease immunity characterised by an overall reduction in the severity of symptoms during successive infections [2, 3]. Adults and older children rarely experience life-threatening severe disease; however, they remain susceptible to infection and to mild disease. Several hypotheses have been advanced to explain the lack of sterilising immunity. The parasite is highly polymorphic and undergoes antigenic variation meaning that the progressive acquisition of immunity depends upon the development of multiple Ab responses [4, 5]. However, it has also been suggested that B-cell memory itself is aberrant after malaria infection, which delays the acquisition of immunity [6].

Individuals exposed to *Plasmodium* infections often carry high numbers of atypical memory B cells (aMBCs) which are CD19^hi^ but CD27^−^ and CD21^−^ [7–12]. Unlike CD27+ CD21+ classical memory B cells (cMBCs), aMBCs do not proliferate, differentiate into Ab-secreting cells or even secrete cytokines upon in vitro stimulation with TLR ligands but express multiple inhibitory receptors [7, 13, 14]. They are often expanded in chronic infections like HIV, and in autoimmune diseases such as systemic lupus erythematosus [15–17] and ageing [18, 19] leading to the hypothesis that they are exhausted B cells associated with poor function and impaired Ab responses. However, it has also been proposed that aMBCs are in fact simply recently activated cells and they are therefore seen in chronic infection and autoimmunity as a result of high Ag burden [20, 21]. In agreement with this, cells expressing the aMBC marker CD11c were found to be expanded during acute malaria [8].

A key outstanding question is whether aMBCs develop in response to high or persistent Ag exposure or form as a result of the inflammatory milieu of malaria infection. To distinguish these possibilities, we used Ag-specific tetramers to examine responses to *Plasmodium falciparum (Pf)* Ags and to tetanus toxoid (TT) in two groups of adults in Kilifi, Kenya: (a) adults with previously high exposure, but no longer exposed to malaria, and (b) adults with continuous high exposure to malaria transmission, to enable us to independently examine the effects of Ag exposure and bystander inflammatory responses associated with persisting high exposure on the formation of aMBCs.

## Results and discussion

### High levels of circulating *Pf*-specific antibodies and MBCs in areas of moderate to high transmission

**Figure 1:**
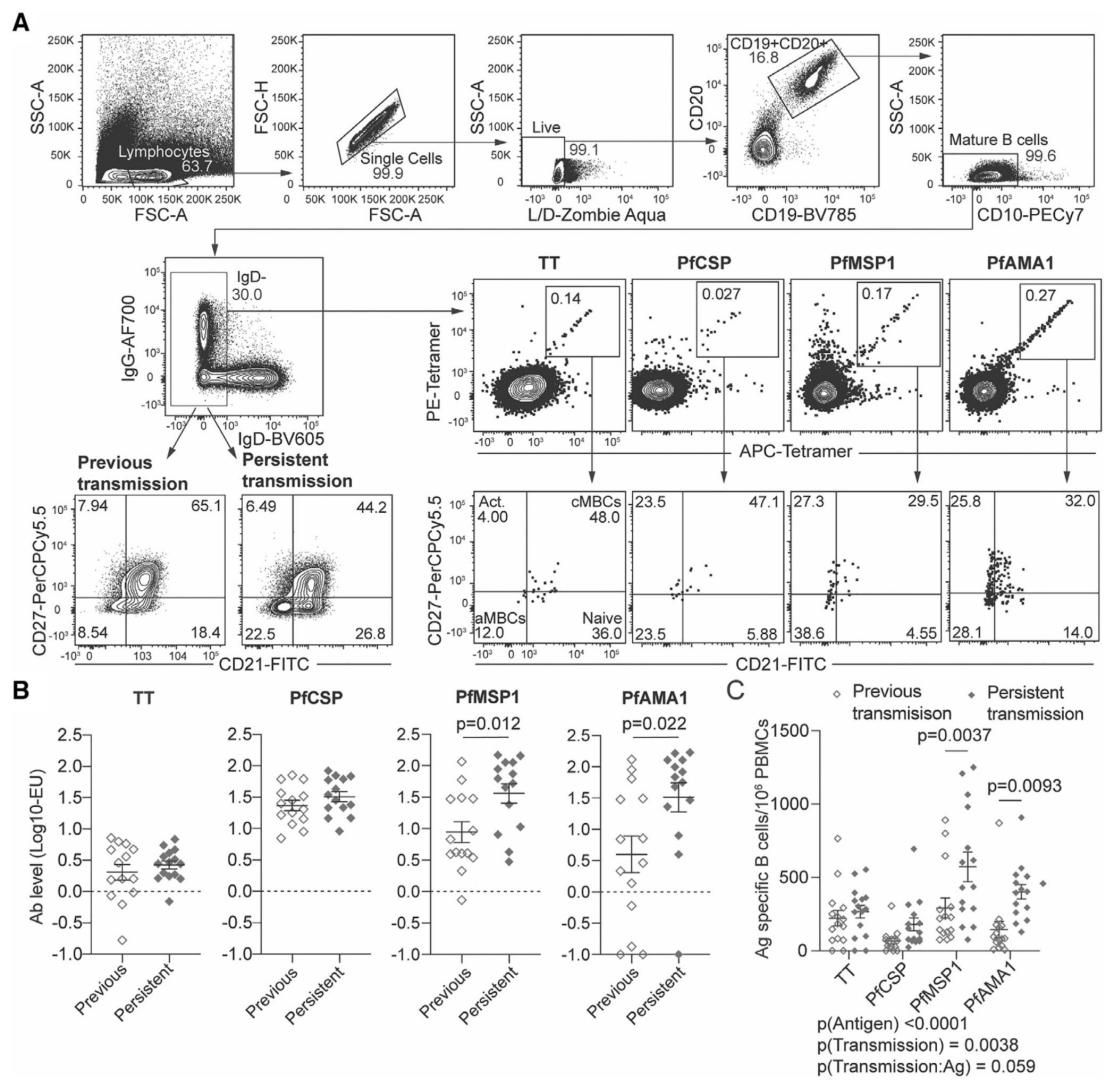
Individuals in areas of persistent transmission have high numbers of circulating B cells specific for blood stage Ags. PBMCs from 30 individuals (15 previously exposed; 15 persistently exposed in one experiment) were analysed to determine the frequency of Ag-specific MBCs and Ab titers to TT and a panel of malaria Ags. (A) General gating strategy for the identification of Ag-specific B cells and determination of B-cell phenotype. Data from a representative individual who had detectable levels of Ag-specific B cells for all Ags studied shown. (B) Quantitation of levels of circulating antibodies to TT, PfCSP, PfMSP1 and PfAMA1, among individuals exposed to moderate or low levels of malaria transmission. Units are international units for TT and arbitrary ELISA units for the *Plasmodium* Ags; mean ± SEM shown, data analysed by Students t-test for each Ag. (C) The number of tetramer^+^ IgD^-^ B cells per million PBMCs specific for each of the Ags studied; mean ± SEM shown; analysis was via two-way ANOVA controlling for subject as a random effect; details of the model are given below the graph.

To track Ab and B-cell responses to *Pf* and non-*Pf* Ags, PBMCs and plasma were taken from two cohorts of adults living in Kilifi, Kenya: (a) adults with previously high exposure but currently low exposure to malaria transmission (previously exposed), and (b) adults with continuous high exposure to malaria transmission throughout their lives (persistently exposed) (Supporting Information Table 1). Ab levels were quantified by ELISA, while Ag-specific activated (IgD^−^) B cells were identified by flow cytometry using fluorescently tagged tetramer probes and phenotyped via staining for CD27 and CD21 (Fig. 1A). We focussed on three *Pf* Ags: the circumsporozoite protein (PfCSP) NANP peptide with nine repeats, which contain the B-cell epitopes for the PfCSP protein coating the surface of the infectious *Pf* sporozoite stage; merozoite surface protein 1 (PfMSP1) which is a blood stage Ag; and apical membrane Ag 1 (PfAMA1) which is expressed in both sporozoites and blood stages. Responses to TT were examined since diphtheria, pertussis, and tetanus mixed vaccine (DTP) vaccination and boosting is widespread in our population.

Analysis of Ab responses revealed that antibodies to the blood stage Ags, PfAMA1 and PfMSP1, but not PfCSP were significantly higher in the persistently exposed compared to previously exposed adults (Fig. 1B). In contrast responses to TT were similar between the persistently and previously exposed adults (Fig. 1B). We further found that frequencies of PfMSP1- and PfAMA1-specific IgD^−^ B cells were significantly higher in persistently exposed compared to previously exposed adults for all the malaria Ags studied, (Fig. 1C). In contrast, the numbers of TT- and PfCSP-specific B cells were similar between persistently and previously exposed adults, matching the Ab data (Fig. 1C).

### The development of atypical B cells is driven by both Ag and bystander effects

**Figure 2:**
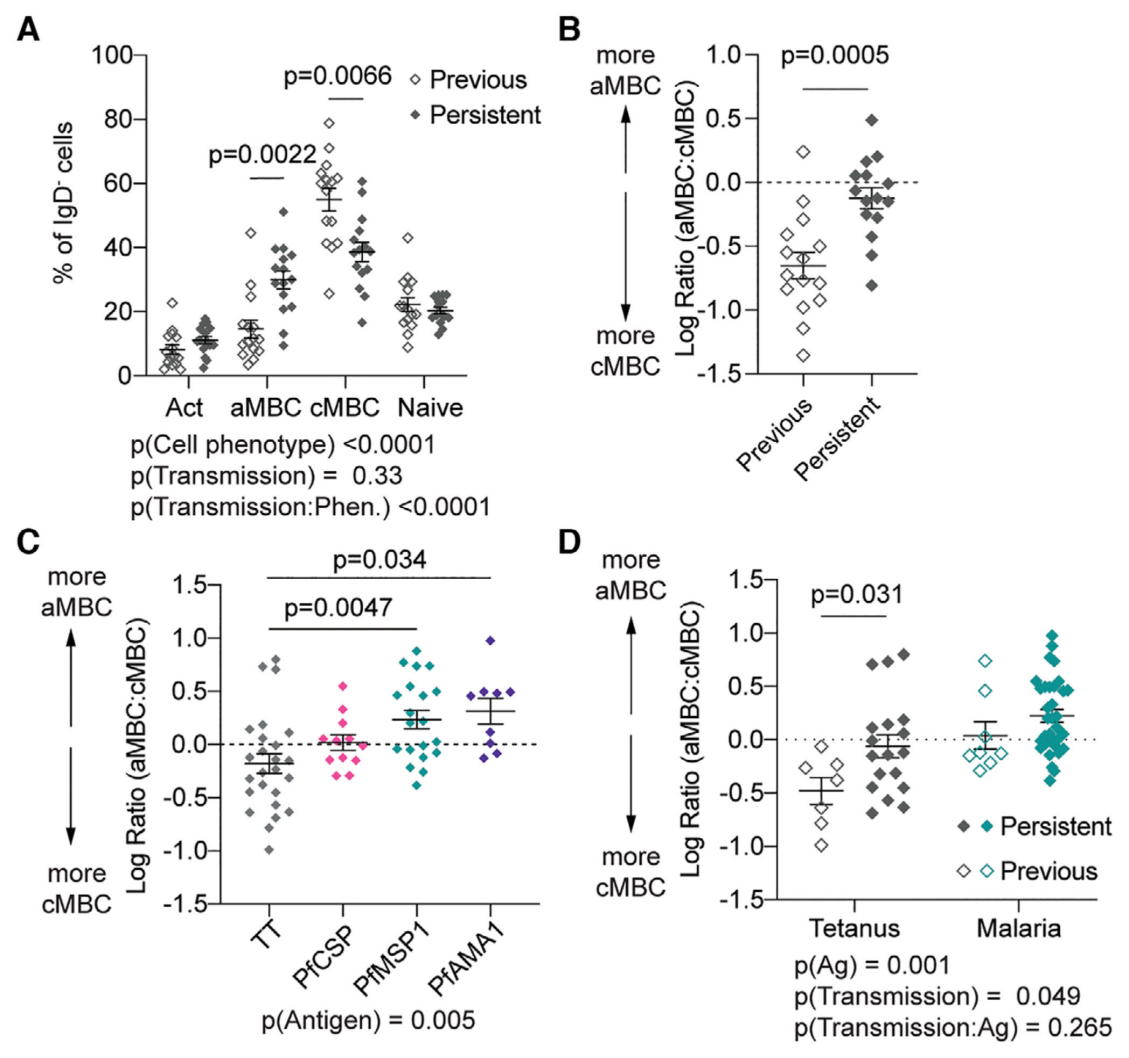
Ag exposure and malaria transmission drive the atypical phenotype in B cells. PBMCs from the 30 individuals (15 previously exposed; 15 persistently exposed in one experiment) described in Fig. 1 were analysed for the phenotype of bulk and Ag-specific B cells. (A) Percentages of bulk IgD^−^ B cells that have the cMBC, aMBC, activated and naïve phenotypes in individuals exposed to moderate and low levels of malaria transmission; mean ± SEM shown; analysis was via two-way ANOVA with Tukey post-test controlling for subject as a random effect; details of the model are given below the graph. (B) Log ratio of aMBC to cMBCs among bulk IgD^−^ B cells in individuals exposed to low and moderate levels of malaria transmission. Mean ± SEM shown; analysis via Student’s t-test. (C) Log ratio of aMBC to cMBCs among Ag-specific B cells pooled from all donors, regardless of transmission status who had detectable levels of circulating Ag-specific B cells. Data are presented as mean ± SEM with analysis via one-way ANOVA with Tukey post-test controlling for subject as a random factor with pairwise comparisons made to the TT (control) group; *n* = 66 observations, observations were only included if there were >10 Ag-specific cells/sample. (D) Log ratio of aMBC to cMBCs among malaria-specific (pooled from PfCSP, PfMSPl and PfAMAl) and TT-specific B cells segregated by the levels of malaria exposure; mean ± SEM shown and analysed via two-way ANOVA with Tukey post-test controlling for subject as a random effect, details of the model are given below the graph.

We further examined the phenotype of bulk and Ag-specific B cells among the individuals in the study via flow cytometry (Fig. 1A). In agreement with previous work we found that persistently exposed adults living with malaria have a higher proportion of CD21^−^, CD27^−^ aMBCs compared to previously exposed adults (Fig. 2A). Correspondingly, persistently exposed adults have a lower proportion of CD21+, CD27+ cMBCs (Fig. 2A). Overall, levels of naïve (CD21+, CD27^−^) and activated (CD21^−^, CD27+) B cells were however similar between the persistently and previously exposed adults (Fig. 2A). Because the differences in overall B-cell phenotypes between the persistently and previously exposed adults were driven by inverse effects on the aMBC and cMBC proportions, we were able to simplify our analysis by focussing on the ratio of these two groups (Fig. 2B).

We next examined the ratio of atypical to classical B cells among Ag-specific B cells. Overall *Pf*-specific B-cell populations included a higher proportion of aMBCs compared to responses to TT (Fig. 2C). Among the malaria Ags it was noticeable that higher proportions of aMBCs were seen in responses to the blood stage Ags, PfMSP1 and PfAMA1, compared to the pre-erythrocytic PfCSP Ag. As few *Pf* sporozoites are injected with each bite, it is likely that overall exposure to this Ag is lower which may explain the lower aMBC response to this Ag. Overall these data indicate that persistent malaria exposure drives cognate B cells to form large populations of aMBCs.

We were further able to address the question of whether persistent malaria transmission drives bystander (non-malaria specific) B cells to develop an atypical phenotype. We compared the ratio of aMBCs to cMBCs B cells in TT- and malaria-specific B cells (pooled responses for the three Ags) and analysed the results by two-way ANOVA to examine the effects of Ag and transmission status. As expected, the effect of Ag specificity remained significant even when transmission status was included in the model (Fig. 2D). More surprising, persistent exposure was associated with a higher proportion of aMBCs among Ag-specific B cells, even when examining TT-specific responses. These findings indicate that malaria exposure can drive bystander B cells to develop aMBC phenotype. One hypothesis is that *Pf* infection can drive high levels of Type 1 cytokines that stimulate the production of Tbet, which is upregulated in aMBCs [22].

### Ag exposure and selection drives different patterns of mutation in *Pf*- and TT-specific B cells

**Figure 3:**
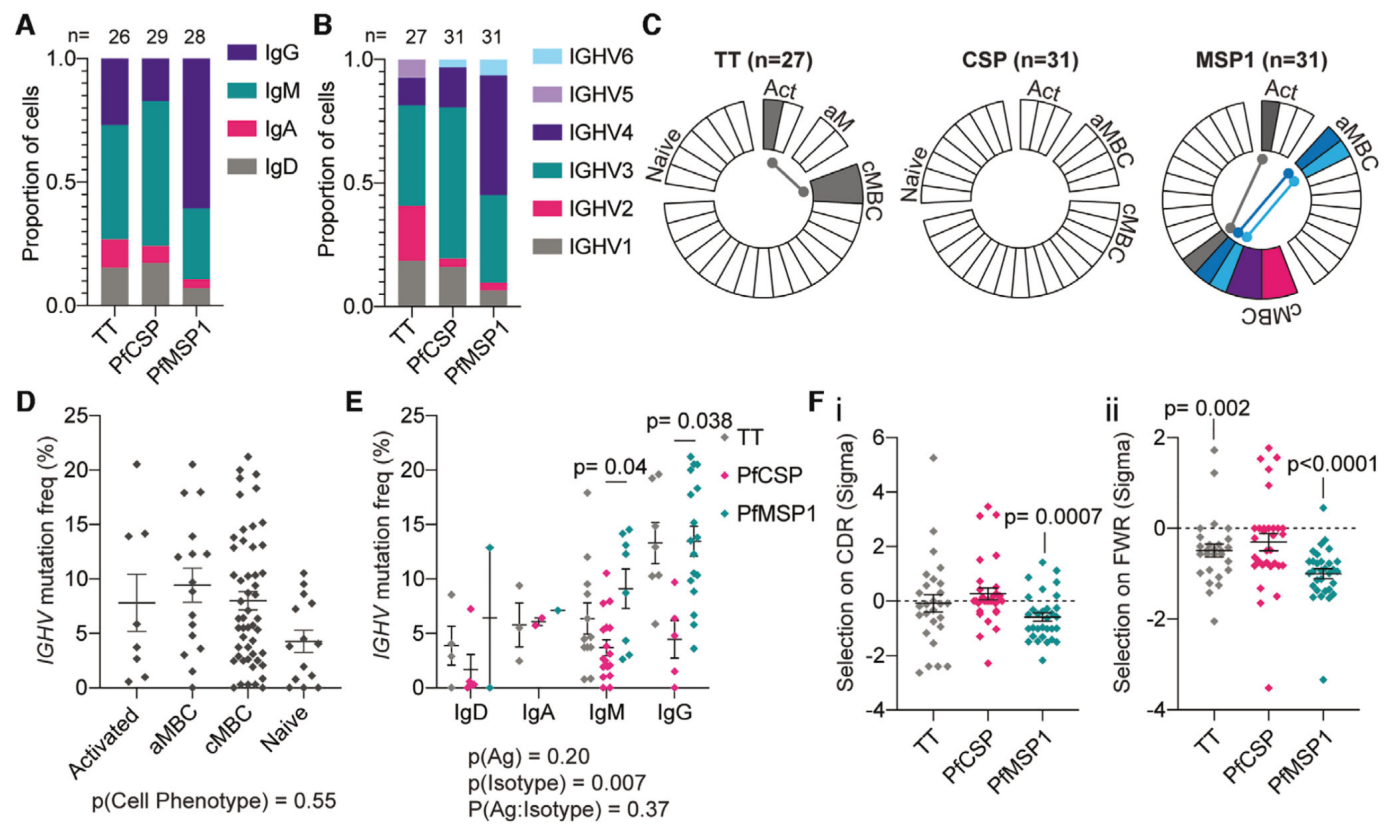
Diversity of Ab responses to TT and *Plasmodium* Ags. B cells specific for TT (*n* = 27 cells from 2 donors), PfCSP (*n* = 31 cells from three donors) and PfMSP1 (n = 31 cells from three donors, one sort per donor) were sorted after tetramer staining and rearranged Ig V(D)J sequences and constant regions determined by RNA-seq. Analysis of (A) Ig isotype and (B) *IGHV* gene use by Ag-specific B cells; note for some cells it was not possible to determine the isotype used. (C) Analysis of clonal relationships between B cells specific for each Ag; each wedge constitutes a unique clone, clones with multiple representatives are coloured and if a clone spans two phenotypes the link is indicated. (D) Analysis of mutation frequency by cell phenotype; bars show mean ± SEM; analysis was by one-way ANOVA with Tukey post-test controlling for subject as a random effect. (E) Association of mutation frequency with Ag and Ab isotype; analysis by two-way ANOVA controlling for subject as a random factor with Tukey post-test, bars show mean ± SEM details of the model are given below the graph, significant pairwise comparisons indicated. (F) Results of BASELINe analysis of selective pressure (sigma) on Ig gene sequences in (i) CDRs and (ii) FWRs; mean ± SEM shown for each Ag, analysis by single sample ANOVA to determine if the selective pressure is significantly different from zero.

Our final analysis was to determine the extent of affinity maturation and selection in the B-cell response to *Pf* Ags and TT. The rearranged Ig V(D)J sequences of individual TT-, PfCSP- and PfMSP1-specific B cells were obtained by single-cell RNA-seq (Supporting Information Table 3). Between B cells specific for the different Ags, there were differences in the isotype (Fig. 3A; *χ* ^2^ (6, *n* = 83) = 13.7, *p* = 0.032) and V region usage (Fig. 3B; *χ* ^2^ (10, *n* = 89) = 27.7, *p* = 0.002). In particular, B cells responding to PfMSP1 were more likely to be IgG+ and use *IGHV4* family genes, while PfCSP-specific B cells mainly used *IGHV3* family genes, consistent with previous findings [23, 24]. Neither Ig isotype (*χ* ^2^ (3, *n* = 61) = 6.89, *p* = 0.076) nor V region usage (*χ* ^2^ (5, *n* = 67) = 9.13, *p* = 0.10) were significantly associated with whether a cell differentiated to become an aMBC or cMBC (Supporting Information Fig. 1A and B), though due to the relatively small sample size we may not have the power to detect differences in this analysis.

Strikingly, we observed some expanded clones in the cells responding to TT and PfMSP1. In particular, five expanded clones from two different individuals were found among the PfMSP1-specific cells and in three cases, the members of the clones had multiple cell fates (cMBC and aMBC, or cMBC and activated) suggesting that individual cells are capable of differentiating into multiple cell fates (Fig. 3C).

Analysis of the frequency and patterns of mutation revealed differences between B cells responding to the different Ags. Simple one-way ANOVA suggested that mutation frequency was not significantly different between cells with different phenotypes (Fig. 3D), and this result did not change when Ab isotype and Ag specificity were included in a more complex linear model (*p* = 0.67 for cell phenotype; *p* < 0.001 for both isotype and Ag specificity). These data are consistent with findings from an analysis of V region usage and SHM in bulk aMBCs and cMBC [25]. On the other hand, both Ab isotype and Ag specificity appeared to be associated with the level of mutation via one-way ANOVA (Supporting Information Fig. 1C and D). However, because our previous analysis revealed that there were significant associations between Ag and isotype (e.g. PfMSP1 cells are more likely to have switched to secreting IgG; Fig. 3A), we analysed the data in a two-way ANOVA model incorporating both factors (Fig. 3E). In this model isotype was the main driver of mutation frequency, however PfCSP-specific IgG^+^ and IgM^+^ cells were still found to be significantly less mutated than PfMSP1-specific IgG^+^ and IgM^+^ cells (Fig. 3E). Others have reported a low mutation frequency in B cells responding to PfCSP and it is unclear why this is, though it has been suggested that affinity maturation to PfCSP is driven more by expansion of high affinity germline clones rather than somatic hypermutation [23, 24].

To understand why PfMSP1-specific cells carried higher levels of mutation, we examined the strength and direction of selective pressure on the rearranged Ig genes specific for the different Ags by comparing the number of synonymous and non-synonymous mutations using BASELINe analysis [26]. Overall there was little evidence of a bias towards positive or negative selection among TT- and PfCSP-specific Ig genes, however PfMSP1-specific BCRs showed strong evidence of negative selection in both the complementarity determining regions (CDRs; Fig. 3Fi) and the framework regions (FWRs; Fig. 3Fii). This could occur if PfMSP1-specific B cells undergo multiple rounds of somatic hypermutation without acquiring any further beneficial (affinity increasing) mutations resulting in the accumulation of silent (synonymous mutations). This would be consistent with these B cells being continuously exposed to their cognate Ag as is likely to occur in conditions of moderate to high malaria transmission where the likelihood of infection is high. Collectively, our analysis of Ig gene usage and mutation frequency reveals that the nature of the Ag and extent of exposure shape the Ab response.

### Concluding remarks

Our analysis reveals that malaria exposure may drive both cognate and non-cognate cells to an aMBC phenotype. Different Ags by virtue of structure and nature of exposure also induce different patterns of mutation with responding Ig genes. The finding that malaria-specific B cells were more likely than TT-specific B cells to become aMBCs suggests that this phenotype may be driven by chronic Ag exposure. However non-specific effects such as inflammation may also drive the formation of atypical cells as even TT-specific B cells were more likely to be atypical in areas of ongoing malaria transmission. The prevalence of atypical TT-specific B cells in the absence of persistent exposure to TT indicates that atypical cells are unlikely to be merely recently activated cells as has been suggested from recent mouse studies [20, 21]. A key outstanding question remains whether the development of aMBCs negatively impacts the overall Ab response to both malaria Ags and bystander vaccine Ags. An alternative hypothesis is that aMBCs are a functional component of diverse MBC response. Addressing these questions will, however, require better tools to measure the function of different MBC subsets in humans.

## Materials and methods

### Ethical approval

This study was approved by the Kenyan Medical Research Institute National Ethics Committee and the Australian National University Human Research Ethics Committee (protocol number 2014/102). Research was conducted according to the principles of the Declaration of Helsinki, which included the administration of informed consenting in the participant’s local language.

### Study population and site

For this study, adults (Supporting Information Table 1) living in two villages 20 km apart from each other in Kilifi, Kenya, with Junju lying on the southern side and Ngerenya on the northern side of an Indian Ocean creek, were recruited. Junju is under continuous moderate to high malaria transmission intensity with *Pf* prevalence at 30% during the dry period between January to May when *Pf* transmission is minimal and up to 70% during the high malaria transmission seasons (May to August and October to December) [27, 28]. In contrast, *Pf* transmission has dramatically reduced in Ngerenya, which was endemic with a parasite prevalence of 40% in 1998, and a transmission intensity of 10 infective bites per person per year [29], to between nil and very low levels since 2005 [7, 30].

Except for the single-cell sorting and sequencing that were done at The Australian National University, all other experiments were performed at the KEMRI/Wellcome Trust Research Laboratories, Kilifi, Kenya.

### Antigens

Ag-specific MBCs and Ab titers were quantified against the NANP peptide (with nine NANP repeats) from the sporozoite stage PfCSP Ag, and recombinant proteins based on three alleles of each of the blood stage Ags: PfAMA1(FVO/3D7/L32) and PfMSP1 42 kDa (FVO/FUP/3D7) (provided by L. H. Miller (National Institutes of Health, Rockville, MD)) mixed at a ratio of 1:1:1. TT (NIBSC, United Kingdom) was used as non-malaria control Ag.

### ELISA

Plasma samples were tested for *Pf* Ag(s) and TT-specific antibodies using a standard ELISA protocol. Immunlon 2 HB plates (Dynatech Laboratories) were coated with 1 μg/mL of each test Ag (100 μL/well) and incubated overnight at 4°C. Non-specific binding sites were blocked with PBS containing 0.1% Tween-20 and 3% skim milk for 5 h at room temperature. The plates were incubated with 100 μL of test plasma (diluted 1:1000 in blocking buffer) overnight at 4°C and then alkaline phosphatase conjugated anti-human IgG (Sigma) for 1 h at room temperature. Plates were washed thrice with PBS containing 0.1% Tween-20 after each incubation step and visualised by adding P-nitrophenyl phosphate (Sigma) substrate. The substrate reaction was stopped with 50 μL/well of 3M sulphuric acid before being read in ODs at 405 nm. Generally, the ELISA backgrounds were very low with the mean ODs for a duplicate blank control included each ELISA plate being 0.06. The ODs for malaria naïve European control plasma samples were 0.087 (0.02 SD, *n* = 10), 0.07 (0.01 SD, *n* = 10) and 0.06 (0.01 SD, *n* = 10) for PfCSP, PfAMA1 and PfMSP1, respectively. In contrast, the average ODs for the malaria test samples were 0.29 (0.10 SD, *n* = 15), 1.99 (0.95 SD, *n* = 15) and 1.41 (0.90 SD, *n* = 15) among the persistently exposed adults, and 0.19 (0.08 SD, *n* = 15), 0.89 (0.84 SD, *n* = 15) and 0.52 (0.50 SD, *n* = 15) among the previously exposed adults, for PfCSP, PfAMA1 and PfMSP1, respectively. The average ODs for TT antibodies were 1.70 (0.95 SD, *n* = 15) and 1.33 (0.92 SD, *n* = 15) among the persistently and previously exposed adults, respectively.

Purified hyperimmune and control human TT IgG were used as standards for *Pf*-specific and TT ELISAs respectively. Ab concentrations for *Pf* Ags and TT were expressed as arbitrary or international units, respectively, determined against the respective standard curves.

### Tetramer preparation and flow cytometry

*Pf*PfMSP1, PfPfAMA1 and TT were biotinylated with the Sulfo-NHS-LC-Biotinylation Kit (ThermoFisher) at a ratio of 1:1 according to the manufacturer’s instructions. For detection of PfCSP-specific cells biotinylated (NANP)_9_ peptide was sourced from Biomatik (Ontario, Canada). Biotinylated Ags were incubated with premium-grade SA-PE and SA-APC (Molecular Probes) at a molar ratio of 4:1, added four times with 15 min incubation at room temperature.

PBMCs, processed and cryopreserved at 5 × 10^6^ [31], were thawed and washed in PBS with 2% heat inactivated FBS. Approximately 2.5 × 10^6^ cells were incubated with 1 μL of both SA-PE and SA-APC tetramers for 20 min at room temperature. Cells were further incubated for an additional 30 min with human B-cell surface molecules using the antibodies listed in Supporting Information Table 2. One million events were acquired on a BD Fortessa flow cytometer (BD Biosciences, San Jose, CA) and the data analysed using FlowJo software (Tree Star, Inc, Ashland, OR).

### Single-cell RNA-seq to sequence recombined Ig V(D)J chains

Lymphocytes were gated using forward scatter (FSC) versus side scatter (SSC) plot followed by doublet and viability discrimination as in Fig. 1A. Singlet lymphocytes were gated on CD19+CD20+CD10^−^ to identify mature B cells and IgD^−^ double tetramer positive Ag-specific single cells were index sorted on FACSAria II (BD Biosciences). Cells were collected into a 96-well plate containing 2 μL lysis buffer (0.2% v/v) Triton X-100, 5 μM oligo-dT primer (Vir70) (both from Sigma), RNase inhibitor (Clontech) and 10 Mm dNTP (Promega), briefly centrifuged and stored at −80°C until further processing. As a positive control for mRNA amplification and PCR processing, ten mature B cells were sorted into three wells and processed in the same way as single cells.

Single-cell RNA sequencing was performed using a SMARTseq 2 protocol [32] with the following modifications. Cells were sorted into plates with wells containing 1 μL of the cell lysis buffer, 0.5 μL dNTP mix (10 mM) and 0.5 μL of the oligo-dT primer at 5 μM. We then reduced the amount reagent used in the following RT and PCR amplification step by half. The concentration of the IS PCR primer was also further reduced to 50 nM. Due to the low transcriptional activity of MBCs, we increased the number of PCR cycles to 28. Sequencing libraries were then prepared using the Nextera XT Library Preparation Kit with the protocol modified by reducing the original volumes of all reagents in the kit by one-fifth. Sequencing was performed on the Illumina NextSeq sequencing platform. To determine the Ag-specific BCR repertoire, we used VDJpuzzle [33] to reconstruct full-length heavy and light chains from each cell. From this we were able to determine V region usage and mutation frequency.

### Statistical analysis

Statistical analysis was performed in GraphPad Prism for simple analyses without blocking factors; all other analyses were performed in R (The R Foundation for Statistical Computing) with details of statistical tests in the relevant figure legends. Only significant *p* values were shown.

## Supplementary Material

Supplementary Information

## Abbreviations

aMBCs: atypical memory B cells
cMBCs: classical memory B cells
*Pf*: Plasmodium falciparum
PfAMA1: *Pf* apical membrane Ag 1
PfCSP: *Pf* circumsporozoite protein
PfMSP1: *Pf* merozoite surface protein 1
TT: tetanus toxoid
