# Supplementary material for: Malaria exposure drives both cognate and bystander human B cells to adopt an atypical phenotype
Source: Eur J Immunol. Author manuscript; Available in PMC 2021 Jul 15. (PMC7611263; doi:10.1002/eji.201948473)
Supplement: Supplementary Information [file EMS129563-supplement-Supplementary_Information.zip › eji4724-sup-0001-suppmat.docx]

**Supplementary Information (Aye *et al.)***

Supplementary Information includes:

Supplementary Table 1: Details of study participants

Supplementary Table 2: Antibodies used in this study

Supplementary Figure 1: Additional analysis of V region usage, isotype and somatic hypermutation by antigen specific B cells

Supplementary Dataset: Heavy and Light chain sequence analysis from single cell RNA-seq including sequences, mutation rates and selection index (sigma).

**Supplementary Table 1: Details of study participants**

| **Area** | **Transmission** | **N** | **Gender (F/M)** | **Age range (Ave)** |
| --- | --- | --- | --- | --- |
| Junju | Continuous | 15 | 6/9 | 21-57 (36.1) |
| Ngerenya | Ceased | 15 | 7/8 | 31-56 (41) |

**Supplementary Table 2: Antibodies used in this study**

| **Marker** | **Fluorochrome** | **Clone** | **Vendor** |
| --- | --- | --- | --- |
| A. B cell Phenotyping Panel | | | |
| CD10 | PE-Cy7 | H110a | Biolegend |
| CD19 | BV785 | HIB19 | Biolegend |
| CD20 | APC-Cy7 | 2H7 | BD Biosciences |
| CD21 | FITC | Bu32 | Biolegend |
| CD27 | PerCP Cy5.5 | M-T271 | Biolegend |
| IgD | BV605 | IA6-2 | Biolegend |
| IgG | AF700 | G18-145 | BD Biosciences |
| Live Dead | Zombie Aqua |  | Biolegend |
| B. Single Cell RNA-seq panel | | | |
| CD10 | BV421 | H110a | Biolegend |
| CD19 | BV605 | SJ25CI | BD Biosciences |
| CD20 | APC-Cy7 | 2H7 | BD Biosciences |
| CD21 | PE-Cy7 | Bu32 | Biolegend |
| CD27 | PerCP Cy5.5 | M-T271 | Biolegend |
| IgD | PE-CF594 | IA6-2 | BD Biosciences |
| IgG | AF700 | G18-145 | BD Biosciences |
| Live Dead | Zombie Aqua |  | Biolegend |


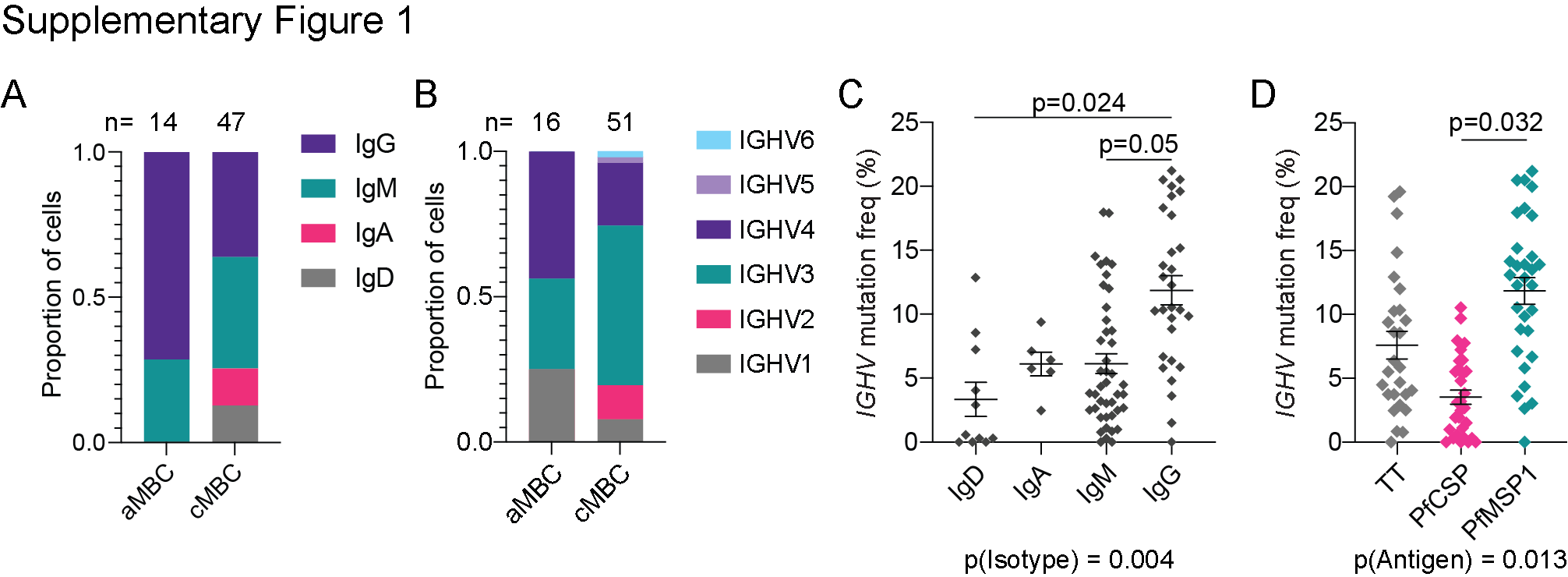


**Supplementary Figure 1: Additional analysis of V region usage, isotype and somatic hypermutation by antigen specific B cells** B cells specific for TT (*n* = 27 cells from 2 donors), PfCSP (*n*= 31 cells from 3 donors) and PfMSP1 (*n* = 31 cells from 3 donors) were sorted after tetramer staining and rearranged Ig V(D)J sequences and constant regions determined by RNA-seq. Analysis of (A) Ig isotype and (B) *IGHV* gene use by antigen specific B cells separated by cell phenotype (cMBC vs aMBC); note for some cells it was not possible to determine the isotype used. C. Analysis of mutation frequency by Ig isotype; bars show mean ± SEM; analysis was by one-way ANOVA with Tukey post-test controlling for subject as a random effect; significant pairwise comparisons indicated. D. Analysis of mutation frequency by antigen; bars show mean ± SEM; analysis was by one-way ANOVA with Tukey post-test controlling for subject as a random effect; significant pairwise comparisons indicated.
